# The impact of the first UK COVID‐19 lockdown on presentations with psychosis to mental health services for older adults: An electronic health records study in South London

**DOI:** 10.1002/gps.5834

**Published:** 2022-11-04

**Authors:** Lauren Simkin, Paul Yung, Flora Greig, Gayan Perera, Konstantinos Tsamakis, Emmanouil Rizos, Robert Stewart, Latha Velayudhan, Christoph Mueller

**Affiliations:** ^1^ Institute of Psychiatry, Psychology and Neuroscience King's College London London UK; ^2^ South London and Maudsley NHS Foundation Trust London UK; ^3^ Second Department of Psychiatry National and Kapodistrian University of Athens ‘Attikon’ University General Hospital Athens Greece

**Keywords:** COVID‐19, delusions, dementia, hallucinations, lockdown, non‐white ethnicity, older adults, psychosis

## Abstract

**Objectives:**

Social distancing restrictions in the COVID‐19 pandemic may have had adverse effects on older adults' mental health. Whereby the impact on mood is well‐described, less is known about psychotic symptoms. The aim of this study was to compare characteristics associated with psychotic symptoms during the first UK lockdown and a pre‐pandemic comparison period.

**Methods:**

In this retrospective observational study we analysed anonymised records from patients referred to mental health services for older adults in South London in the 16‐week period of the UK lockdown starting in March 2020, and in the comparable pre‐pandemic period in 2019. We used logistic regression models to compare the associations of different patient characteristics with increased odds of presenting with any psychotic symptom (defined as hallucinations and/or delusion), hallucinations, or delusions, during lockdown and the corresponding pre‐pandemic period.

**Results:**

1991 referrals were identified. There were fewer referrals during lockdown but a higher proportion of presentations with any psychotic symptom (48.7% vs. 42.8%, *p* = 0.018), particularly hallucinations (41.0% vs. 27.8%, *p* < 0.001). Patients of non‐White ethnicity (adjusted odds ratio (OR): 1.83; 95% confidence interval (CI): 1.13–2.99) and patients with dementia (adjusted OR: 3.09; 95% CI: 1.91–4.99) were more likely to be referred with psychotic symptoms during lockdown. While a weaker association between dementia and psychotic symptoms was found in the pre‐COVID period (adjusted OR: 1.55; 95% CI: 1.19–2.03), interaction terms indicated higher odds of patients of non‐White ethnicity or dementia to present with psychosis during the lockdown period.

**Conclusions:**

During lockdown, referrals to mental health services for adults decreased, but contained a higher proportion with psychotic symptoms. The stronger association with psychotic symptoms in non‐White ethnic groups and patients with dementia during lockdown suggests that barriers in accessing care might have increased during the COVID‐19 pandemic.

## Key points

- Referrals to mental health of older adult services during lockdown were a third of the pre‐pandemic period, but the proportion with any psychotic symptom was higher.
- Non‐White ethnicity was associated with any psychotic symptom during the lockdown, but not in the pre‐COVID comparison period.
- A referral with a diagnosis of dementia was more likely to be associated with any psychotic symptom, predominantly hallucinations, during the lockdown period.
- Inequalities in access to care, already present before the pandemic, may have been exacerbated during lockdown, leading to fewer referrals and more severe presentations with psychosis.

## 1 INTRODUCTION

Social distancing restrictions implemented by countries around the world to reduce the spread of coronavirus disease 2019 (COVID‐19) have been found to be associated with deteriorating mental health of the general population.^1^ The effects might have an even higher impact on older people for a number of reasons, including fear related to higher morbidity and mortality, reduction in already restricted means of socialising (e.g., closed community centres or places of worship), and difficulties in accessing modern technologies to remain in contact with relatives.^1^, ^2^, ^3^, ^4^, ^5^

Evidence has already emerged of an increase in affective symptoms in older adults during the pandemic,^2^, ^6^, ^7^ particularly in those living alone and therefore perhaps more vulnerable to the impact of social distancing measures. However, little is known about how lockdown affects psychotic symptoms in the older adult population.^8^

A number of features of the lockdown may increase the risk of developing or exacerbating a psychotic illness. High perceived stress, as a result of fears generated during the pandemic, can be an important risk factor for both triggering and exacerbating psychotic symptoms.^9^ In addition, lockdown has been associated with social isolation and loneliness^10^, ^11^ and several case reports have described new onset psychosis in older adults attributed to social isolation.^12^, ^13^, ^14^

Given the complexities of diagnosing and treating late‐life psychosis,^15^ understanding the impact of the pandemic on the older adult population is essential to inform care. Although for many countries social distancing measures have been stopped, for others, they remain a part of life. We sought to understand this subject further through analysing data on routine referrals to mental health of older adult services (MHOA) of a large mental health trust during the first UK lockdown and before the pandemic.

## 2 METHODS

### 2.1 Data source

We carried out a retrospective observational study using anonymised electronic healthcare records from the South London and Maudsley NHS Foundation Trust (SLaM). SLaM is one of the largest specialist mental healthcare providers in Europe, serving a population of approximately 1.36 million residents across four South London boroughs (Lambeth, Lewisham, Croydon, and Southwark). SLaM's de‐identified electronic health records can be accessed for research purposes via the Clinical Record Interactive Search (CRIS) platform.^16^ CRIS was developed in 2007–2008, has supported over 250 publications, and is approved by the Oxford Research Ethics Committee C (reference 18/SC/0372) as a database for secondary analysis.^16^ Data are extracted both from structured fields and from free text (e.g., clinical case notes, correspondence), the latter through natural language processing (NLP) algorithms developed using General Architecture for Text Engineering (GATE) software.^17^, ^18^

### 2.2 Study sample

All accepted referrals to SLaM's older adult community and general hospital liaison services were identified during the first UK COVID lockdown (16/3/2020–5/7/2020, 16 weeks) and the equivalent period in the preceding year (18/03/2019–7/7/2019, 16 weeks). Date of referral was defined as index date for definition of patient characteristics.

### 2.3 Outcomes: Psychotic symptoms

The primary outcome variable was the presence of psychotic symptoms around the index date (maximum of 6 months before or after) in the electronic health record. We ascertained whether patients presented with either hallucinations (regardless of whether patients also had delusions), delusions (regardless of whether patients also had hallucinations), or both (referred to as any psychotic symptom) using NLP to identify documentation of these symptoms from free text.^16^ The NLP algorithm has previously been evaluated and in a random sample of 100 health‐care documents, whereby high accuracy in identifying delusions (pre‐annotated documents: precision = 90%; un‐annotated documents: precision = 93%, recall 85%) and hallucinations (pre‐annotated documents: precision = 90%; un‐annotated documents: precision = 84%, recall = 98%) was demonstrated.^19^

### 2.4 Factors potentially associated with psychosis

We additionally extracted data on a number of clinical and sociodemographic characteristics, including age at referral, gender, and ethnicity (dichotomised to White and non‐White). We further characterised the presence of the following mental health diagnoses according to ICD‐10 criteria^20^ closest to the index date: dementia (F00‐F03), psychotic illness (F20‐29), affective disorder (F30‐F39), and delirium (F05).

The presence and severity of clinical symptoms related to the referral, including mental and physical health problems and functional difficulties, were estimated from the Health of the Nation Outcome Scales (HoNOS) which are routinely completed in SLaM patients.^21^ HoNOS items we included in our analysis were the mental health problems of agitated behaviour, non‐accidental self‐injury, problematic substance or alcohol use, cognitive difficulties, depressed mood, physical illness/disability, as well as functional impairment, reflected in difficulties with activities of daily living (ADL), problems with relationships (social impairment), problems with living condition, and problems with daytime activities. These subscales are rated from 0 (no problem) to 4 (severe or very severe problem). We dichotomised these scores to define binary variables: 0–1, ‘minor or no problems’ and 2–4, ‘mild to severe problems’.^21^

Through established NLP algorithms we further identified whether patients were living alone, experiencing disturbed sleep and were prescribed psychotropic medications (antidepressants, antipsychotics, mood stabilisers or sedative medications) within a window from 6 months before or to 6 months after referral.^18^

### 2.5 Statistical analysis

All statistical analyses were carried out using STATA version 15 (StataCorp. 2017. Stata Statistical Software: Release 15. StataCorp LLC.). Initially, descriptive statistics were generated to compare those referred during lockdown and the corresponding pre‐COVID period. Next, logistic regression models were assembled to investigate associations of sociodemographic characterisitics, diagnosis (present vs. not present), mental or physical health problems, functional difficulties and pharmacotherapy as independent variables, and psychotic symptoms (any psychotic symptom, hallucinations, delusions) as the dependent variable, during lockdown and the corresponding pre‐COVID period. For each time period we applied an unadjusted logistic regression model, and a model adjusted for age, gender, ethnicity, and diagnosis. Lastly, we calculated the interaction between the individual clinical characteristic and the presence of a lockdown in predicting the respective psychotic symptom. Because 36% of referrals had missing data on at least one covariate and we judged missingness to be at random, we generated 36 imputed datasets through replacing missing values with simulated values gathered from covariates and outcome values.^22^

## 3 RESULTS

We identified 1991 older adults with accepted referrals to the MHOA services within the first lockdown period (16/03/2020–05/07/2020) and comparable pre‐COVID period (18/03/2019–07/07/2019) combined. Of these, 1455 (73%) occurred during the pre‐COVID period and 536 (27%) during the lockdown period, indicating a significant drop in referrals in the early phase of the pandemic.

Table 1 summarises the demographic and clinical characteristics of the full sample and by each referral period. In the full sample the mean age (SD) at referral was 77.9 (±9.5) years and 56.9% of patients were female. While there were no significant differences in age and gender between the referral periods, the proportion from non‐White ethnicity backgrounds, with dementia, and living alone were lower during the lockdown period. Patients referred during lockdown more frequently had a diagnosis of affective disorder or delirium. Agitated behaviour, non‐accidental self‐injury, disturbed sleep, physical health problems as well as impairments of social functioning were more common during lockdown, but no differences in problems with activities of daily living were found. Use of antipsychotics, antidepressants, mood stabilisers and sedative medications were all recorded in a significantly higher proportion of referrals during the lockdown period than the pre‐COVID period.

**TABLE 1:** Sample characteristics of the full cohort, referrals during lockdown and the corresponding same period pre‐COVID

Characteristics	Full cohort (n = 1991)	Lockdown period (n = 536)	Pre‐COVID period (n = 1455)	*P*‐value^a^
Socio‐demographic status^b^				
Mean age at referral (SD)	77.9 (9.5)	78.4 (8.4)	77.7 (9.8)	0.135
Female gender (%)	56.9	54.9	57.7	0.261
Non‐White ethnicity (%)	28.4	23.8	30.0	0.014
Diagnosis				
Dementia (%)	38.2	31.2	40.8	<0.001
Psychotic illness (%)	6.5	7.7	6.1	0.220
Affective disorder (%)	15.3	18.1	14.3	0.037
Delirium (%)	9.8	14.9	8.0	<0.001
HoNOS symptoms/disorders^b^				
Agitated behaviour (%)	19.7	26.3	17.7	<0.001
Non‐accidental self‐injury (%)	5.4	9.0	4.3	0.001
Problem‐drinking or drug taking (%)	5.4	7.0	4.8	0.108
Cognitive problems (%)	56.1	56.5	56.0	0.885
Depressed mood (%)	28.4	31.8	27.3	0.096
Physical illness or disability (%)	60.6	65.8	59.0	0.022
HoNOS functional problems^b^				
Activities of daily living (%)	52.0	55.3	50.9	0.146
Living conditions (%)	14.9	22.0	12.7	<0.001
Daytime activities (%)	32.9	39.4	30.9	0.003
Social relationships (%)	21.0	27.8	18.8	<0.001
Other contextual factors^c^				
Living alone (%)	48.9	41.6	51.6	<0.001
Disturbed sleep (%)	53.3	59.7	50.9	0.001
Pharmacotherapy^c^				
Antipsychotic (%)	24.7	35.5	20.7	<0.001
Antidepressant (%)	45.8	51.3	43.7	0.003
Mood stabiliser (%)	5.5	7.8	4.6	0.005
Sedative medication (%)	28.9	39.6	25.0	<0.001
Psychotic symptoms^c^				
Any psychotic symptom (%)	44.4	48.7	42.8	0.018
Hallucinations (%)	31.4	41.0	27.8	<0.001
Delusions (%)	27.4	30.2	26.3	0.083

^a^ *t*‐test or *χ* ^2^ test.

^b^ At the time of referral.

^c^ In a 6 months' window around referral date; Lockdown period is defined as 16/03/2020–05/07/2020; Pre‐COVID period is defined as 18/03/2019–07/07/2019.

In the full sample, any psychotic symptom was found in 44.4% of referrals. During the lockdown period there was a significantly higher percentage of referrals associated with any psychotic symptom (48.7%) than in the pre‐COVID period (42.8%). There was no significant difference between the percentage of referrals with delusions in during the first UK lockdown compared with the pre‐COVID period, but the proportion of referrals with hallucinations was about 50% higher in the lockdown compared to the pre‐COVID period.

### 3.1 Factors associated with any psychotic symptoms during the lockdown and pre‐COVID periods

Table 2 shows regression models of patient characteristics associated with having any psychotic symptom (hallucinations, delusions, or both) in the lockdown and the comparable pre‐COVID period, in unadjusted and adjusted logistic regression models.

**TABLE 2:** Associations of demographics, mental and physical health problems, functioning, and pharmacotherapy with the presence of any psychotic symptom (hallucinations and/or delusions)

Clinical characteristics	Unadjusted logistic regression models—Odds ratios (95% CI)				Adjusted logistic regression models—Odds ratios (95% CI)			
	Pre‐Covid	Lockdown	Lockdown × characteristic interaction	P (interaction term)	Pre‐COVID period	Lockdown period	Lockdown × characteristic interaction	P (interaction term)
Socio‐demographic status^a^								
Age above 78	1.03 (0.83–1.26)	0.95 (0.68–1.34)	0.93 (0.63–1.38)	0.722	1.10 (0.88–1.38)	0.89 (0.61–1.30)	0.94 (0.62–1.41)	0.762
Female gender	0.93 (0.75–1.14)	0.91 (0.65–1.28)	0.98 (0.66–1.46)	0.927	0.88 (0.71–1.09)	0.96 (0.67–1.39)	1.05 (0.69–1.59)	0.818
Non‐White ethnicity	1.02 (0.79–1.31)	**2.00 (1.28–3.14)**	**1.97 (1.18–3.26)**	**0.009**	0.89 (0.68–1.16)	**1.83 (1.13–2.99)**	**2.13 (1.26–3.62)**	**0.005**
Diagnosis^a^								
Dementia	1.02 (0.83–1.27)	**1.56 (1.08–2.25)**	1.52 (0.99–2.32)	0.053	**1.55 (1.19–2.03)**	**3.09 (1.91–4.99)**	**1.65 (1.07–2.54)**	**0.024**
Psychotic illness	**5.83 (3.44–9.90)**	**11.19 (3.93–31.87)**	1.92 (0.59–6.20)	0.276	**8.23 (4.71–14.40)**	**20.12 (6.73–60.17)**	1.90 (0.59–6.15)	0.284
Affective disorder	1.15 (0.86–1.54)	0.89 (0.57–1.39)	0.78 (0.46–1.32)	0.353	**1.56 (1.02–2.37)**	**2.11 (1.23–3.62)**	0.80 (0.47–1.36)	0.402
Delirium	1.06 (0.72–1.55)	1.06 (0.66–1.71)	1.01 (0.55–1.85)	0.981	**1.56 (0.27–1.64)**	**2.44 (1.38–4.33)**	1.03 (0.56–1.90)	0.931
HoNOS symptoms/disorders^a^								
Agitated behaviour	**1.57 (1.16–2.12)**	**1.92 (1.21–3.05)**	1.23 (0.72–2.10)	0.453	**1.46 (1.07–1.99)**	**1.74 (1.05–2.88)**	1.25 (0.72–2.18)	0.424
Non‐accidental self‐injury	1.00 (0.58–1.73)	1.29 (0.63–2.65)	1.28 (0.52–3.18)	0.588	1.03 (0.59–1.82)	1.71 (0.79–3.73)	1.33 (0.53–3.29)	0.543
Problem‐drinking or drug taking	0.74 (0.42–1.29)	0.70 (0.31–1.54)	0.94 (0.38–2.36)	0.900	0.64 (0.36–1.16)	0.71 (0.29–1.73)	1.06 (0.40–2.83)	0.900
Cognitive problems	0.91 (0.72–1.15)	1.16 (0.79–1.71)	1.28 (0.81–2.02)	0.283	0.96 (0.73–1.27)	1.02 (0.62–1.69)	1.33 (0.83–2.13)	0.242
Depressed mood	0.94 (0.73–1.21)	1.04 (0.68–1.60)	1.10 (0.67–1.83)	0.698	0.92 (0.69–1.23)	1.29 (0.77–2.15)	1.14 (0.68–1.92)	0.625
Physical illness or disability	1.02 (0.81–1.28)	1.15 (0.77–1.73)	1.13 (0.71–1.80)	0.596	1.03 (0.81–1.32)	1.24 (0.79–1.94)	1.16 (0.82–1.87)	0.542
HoNOS functional problems^a^								
Activities of daily living	1.21 (0.96–1.53)	0.93 (0.61–1.40)	0.77 (0.49–1.21)	0.253	1.18 (0.92–1.51)	0.90 (0.56–1.43)	0.82 (0.51–1.31)	0.404
Living conditions	**1.51 (1.08–2.12)**	1.24 (0.77–2.00)	0.82 (0.46–1.47)	0.509	1.35 (0.95–1.92)	1.13 (0.67–1.91)	0.85 (0.47–1.57)	0.612
Daytime activities	**1.71 (1.34–2.18)**	1.22 (0.83–1.80)	0.71 (0.45–1.13)	0.149	**1.63 (1.27–2.10)**	1.18 (0.76–1.82)	0.75 (0.46–1.20)	0.227
Social relationships	**1.49 (1.12–1.99)**	**1.64 (1.05–2.57)**	1.10 (0.65–1.86)	0.719	1.33 (0.98–1.79)	1.51 (0.93–2.44)	1.16 (0.67–2.00)	0.590
Other contextual factors^b^								
Living alone	**1.37 (1.11–1.69)**	1.22 (0.86–1.72)	0.88 (0.59–1.33)	0.565	**1.47 (1.18–1.83)**	1.10 (0.76–1.60)	0.76 (0.50–1.16)	0.204
Disturbed sleep	**1.75 (1.42–2.15)**	**2.70 (1.89–3.86)**	**1.55 (1.02–2.34)**	**0.040**	**1.73 (1.39–2.17)**	**2.90 (1.94–4.33)**	1.49 (0.97–2.28)	0.070
Pharmacotherapy^b^								
Antipsychotic	**3.85 (2.93–5.05)**	**5.00 (3.39–7.39)**	1.30 (0.81–2.09)	0.277	**3.09 (2.30–4.14)**	**3.94 (2.60–5.96)**	1.38 (0.85–2.25)	0.190
Antidepressant	1.22 (0.99–1.50)	0.84 (0.60–1.18)	0.69 (0.46–1.03)	0.067	**1.30 (1.03–1.64)**	0.91 (0.62–1.33)	**0.65 (0.43–0.99)**	**0.043**
Mood stabiliser	**2.05 (1.24–3.38)**	1.79 (0.94–3.42)	0.87 (0.39–1.98)	0.745	**1.94 (1.15–3.29)**	2.01 (0.99–4.07)	0.88 (0.38–2.02)	0.759
Sedative medication	**1.90 (1.50–2.42)**	**2.34 (1.64–3.33)**	1.23 (0.80–1.88)	0.350	**1.91 (1.49–2.45)**	**2.51 (1.70–3.70)**	1.20 (0.77–1.86)	0.425

*Note*: Adjusted models are adjusted for age, gender, ethnicity and diagnosis; bold = *p* < 0.05.

^a^ At the time of referral.

^b^ In a 6 months' window around referral date.

In the adjusted model (adjusted for age, gender, ethnicity, and diagnosis), clinical characteristics associated with any psychotic symptom regardless of time period referred were: diagnosis of dementia, psychotic illness, affective disorder and delirium, the mental health symptoms of agitated behaviour and disturbed sleep, and the use of antipsychotic and sedative medication.

For three characteristics, interaction terms between lockdown and clinical characteristic were significant, indicating that associations with any psychotic symptom differed between the two time periods. Non‐White ethnicity was only associated with any psychotic symptom during the lockdown, but not the pre‐COVID period, and a referral with a diagnosis of dementia was more likely to be associated with any psychotic symptom during the lockdown than during the pre‐COVID period. While antidepressant prescription was associated with any psychotic symptom during the pre‐COVID period, this wasn't the case during lockdown.

### 3.2 Factors associated with hallucinations during the lockdown and pre‐COVID periods

Table 3 shows patient characteristics associated with being referred to MHOA services with hallucinations. In summary, similar associations were observed to those with any psychotic symptom. Interaction terms with time period indicated that associations of hallucinations with non‐White ethnic background and dementia diagnosis were stronger in the lockdown period, while those with affective disorder and antidepressant use were stronger in the pre‐COVID period.

**TABLE 3:** Associations of demographics, mental and physical health problems, functioning, and pharmacotherapy with the presence of hallucinations

Clinical characteristics	Unadjusted logistic regression models—Odds ratios (95% CI)				Adjusted logistic regression models—Odds ratios (95% CI)			
	Pre‐Covid	Lockdown	Lockdown × characteristic interaction	P (interaction term)	Pre‐Covid	Lockdown	Lockdown × characteristic interaction	P (interaction term)
Socio‐demographic status^a^								
Age above 78	1.13 (0.90–1.42)	0.98 (0.70–1.39)	0.87 (0.58–1.32)	0.521	1.23 (0.96–1.58)	0.89 (0.61–1.30)	0.87 (0.57–1.33)	0.521
Gender	0.98 (0.78–1.23)	0.84 (0.59–1.18)	0.86 (0.56–1.30)	0.466	0.93 (0.73–1.18)	0.86 (0.60–1.25)	0.88 (0.58–1.36)	0.576
Non‐White ethnicity	1.04 (0.80–1.37)	**1.75 (1.13–2.70)**	**1.68 (1.01–2.78)**	**0.045**	0.98 (0.74–1.31)	1.56 (0.98–2.48)	**1.74 (1.03–2.95)**	**0.040**
Diagnosis^a^								
Dementia	0.95 (0.75–1.20)	**1.73 (1.20–2.51)**	**1.82 (1.17–2.82)**	**0.007**	**1.67 (1.22–2.29)**	**3.42 (2.08–5.62)**	**2.02 (1.29–3.17)**	**0.002**
Psychotic illness	**3.84 (2.48–5.94)**	**5.02 (2.41–10.47)**	1.31 (0.56–3.08)	0.538	**6.56 (4.00–10.75)**	**9.52 (4.22–21.50)**	1.31 (0.56–3.10)	0.534
Affective disorder	**1.42 (1.04–1.94)**	0.78 (0.49–1.22)	**0.55 (0.31–0.95)**	**0.032**	**2.47 (1.69–3.60)**	**1.94 (1.10–3.42)**	**0.55 (0.32–0.96)**	**0.037**
Delirium	**1.59 (1.07–2.36)**	1.14 (0.71–1.84)	0.72 (0.38–1.33)	0.294	**2.63 (1.67–4.15)**	**2.38 (1.47–4.78)**	0.71 (0.38–1.34)	0.292
HoNOS symptoms/disorders^a^								
Agitated behaviour	**2.06 (1.51–2.81)**	**2.01 (1.27–3.18)**	0.98 (1.51–2.81)	0.926	**1.90 (1.38–2.62)**	**1.74 (1.07–2.82)**	1.02 (0.59–1.76)	0.940
Non‐accidental self‐injury	1.57 (0.89–2.74)	1.28 (0.63–2.61)	0.82 (0.33–2.03)	0.667	1.59 (0.88–2.87)	1.76 (0.81–3.80)	0.87 (0.35–2.18)	0.766
Problem‐drinking or drug taking	1.08 (0.60–1.98)	0.81 (0.35–1.88)	0.75 (0.28–1.99)	0.560	1.03 (0.55–1.94)	0.82 (0.32–2.08)	0.82 (0.29–2.33)	0.715
Cognitive problems	0.99 (0.77–1.29)	1.29 (0.87–1.91)	1.30 (0.82–2.06)	0.268	1.07 (0.79–1.46)	1.06 (0.64–1.77)	1.31 (0.81–2.12)	0.269
Depressed mood	1.17 (0.89–1.54)	1.11 (0.72–1.71)	0.95 (0.57–1.59)	0.842	1.15 (0.84–1.58)	1.49 (0.90–2.47)	0.98 (0.58–1.66)	0.937
Physical illness or disability	**1.64 (1.25–2.15)**	1.23 (0.81–1.86)	0.75 (0.46–1.21)	0.236	**1.62 (1.22–2.15)**	1.32 (0.83–2.09)	0.74 (0.45–1.23)	0.246
HoNOS functional problems^a^								
Activities of daily living	**1.81 (1.39–2.35)**	1.06 (0.71–1.57)	**0.59 (0.37–0.93)**	**0.023**	**1.71 (1.30–2.26)**	1.03 (0.66–1.59)	0.62 (0.38–1.00)	0.050
Living conditions	**1.60 (1.11–2.30)**	1.17 (0.73–1.88)	0.73 (0.41–1.32)	0.304	1.45 (0.99–2.11)	1.07 (0.64–1.79)	0.76 (0.41–1.40)	0.381
Daytime activities	**1.84 (1.42–2.39)**	1.38 (0.93–2.06)	0.75 (0.47–1.20)	0.228	**1.70 (1.30–2.24)**	1.32 (0.86–2.02)	0.79 (0.49–1.28)	0.336
Social relationships	**1.85 (1.37–2.50)**	1.54 (0.99–2.40)	0.83 (0.49–1.41)	0.498	**1.67 (1.22–2.29)**	1.39 (0.87–2.23)	0.88 (0.51–1.52)	0.644
Other contextual factors^b^								
Living alone	**0.74 (0.59–0.93)**	1.19 (0.84–1.69)	**1.61 (1.06–2.44)**	**0.026**	0.80 (0.63–1.01)	1.08 (0.75–1.57)	1.41 (0.92–2.17)	0.118
Disturbed sleep	**2.35 (1.85–2.99)**	**3.23 (2.22–4.72)**	1.38 (0.88–2.15)	0.162	**2.39 (1.85–3.08)**	**3.78 (2.48–5.76)**	1.38 (0.87–2.20)	0.165
Pharmacotherapy^b^								
Antipsychotic	**3.77 (2.89–4.92)**	**4.54 (3.11–6.62)**	1.20 (0.76–1.91)	0.429	**2.98 (2.22–4.00)**	**3.69 (2.47–5.52)**	1.25 (0.78–2.01)	0.347
Antidepressant	**1.74 (1.38–2.19)**	0.89 (0.63–1.25)	**0.51 (0.34–0.77)**	**0.001**	**1.94 (1.50–2.51)**	1.01 (0.69–1.49)	**0.46 (0.30–0.72)**	**0.001**
Mood stabiliser	**2.33 (1.42–3.83)**	1.83 (0.97–3.44)	0.78 (0.35–1.75)	0.548	**2.19 (1.29–3.72)**	**2.17 (1.09–4.36)**	0.75 (0.33–1.70)	0.485
Sedative medication	**2.41 (1.88–3.10)**	**2.56 (1.79–3.65)**	1.06 (0.68–1.64)	0.798	**2.33 (1.79–3.03)**	**2.75 (1.86–4.05)**	1.07 (0.68–1.69)	0.759

*Note*: Adjusted models are adjusted for age, gender, ethnicity and diagnosis; bold = *p* < 0.05.

^a^ At the time of referral.

^b^ In a 6 months' window around referral date.

### 3.3 Factors associated with delusions during the lockdown and pre‐COVID periods

Table 4 shows patient characteristics associated with being referred with delusions specifically. In the adjusted model, the following factors were associated with delusions in both time periods: diagnoses of dementia and a psychotic illness, disturbed sleep, and the use of antipsychotic, mood stabilising and sedative medications. While affective disorders and delirium were associated with delusions only during the lockdown, but not the pre‐COVID period, the interaction term was not significant. Significant interactions were found for ethnicity, living alone and disturbed sleep: stronger associations of non‐White ethnicity and disturbed sleep with delusions during lockdown, but stronger associations of living alone with delusion pre‐COVID.

**TABLE 4:** Associations of demographics, mental and physical health problems, functioning, and pharmacotherapy with the presence of delusions

Clinical characteristics	Unadjusted logistic regression models—Odds ratios (95% CI)				Adjusted logistic regression models—Odds ratios (95% CI)			
	Pre‐Covid	Lockdown	Lockdown × characteristic interaction	P (interaction term)	Pre‐Covid	Lockdown	Lockdown × characteristic interaction	P (interaction term)
Socio‐demographic status^a^								
Age above 78	0.84 (0.67–1.06)	0.80 (0.55–1.16)	0.95 (0.62–1.48)	0.836	0.94 (0.73–1.21)	0.80 (0.53–1.21)	0.96 (0.61–1.50)	0.845
Gender	0.89 (0.71–1.13)	0.94 (0.65–1.35)	1.05 (0.68–1.63)	0.833	0.85 (0.67–1.09)	1.07 (0.71–1.61)	1.19 (0.75–1.88)	0.450
Non‐White ethnicity	1.30 (0.99–1.69)	**2.24 (1.43–3.50)**	**1.73 (1.03–2.91)**	**0.040**	1.08 (0.82–1.44)	**2.09 (1.28–3.40)**	**1.92 (1.11–3.32)**	**0.020**
Diagnosis^a^								
Dementia	0.97 (0.76–1.22)	1.20 (0.81–1.78)	1.24 (0.79–1.97)	0.350	**1.40 (1.02–1.89)**	**2.68 (1.53–4.70)**	1.31 (0.81–2.10)	0.269
Psychotic illness	**5.99 (3.81–9.43)**	**9.98 (4.64–21.48)**	1.67 (0.68–4.06)	0.261	**7.02 (4.26–11.56)**	**17.3 (7.25–41.4)**	1.65 (0.68–4.04)	0.271
Affective disorder	0.92 (0.66–1.29)	1.04 (0.65–1.68)	1.13 (0.63–2.02)	0.684	1.27 (0.70–1.89)	**2.46 (1.32–4.59)**	1.16 (0.64–2.10)	0.621
Delirium	0.79 (0.51–1.25)	0.80 (0.47–1.36)	1.00 (0.50–2.02)	0.999	1.15 (0.87–1.89)	**1.98 (0.06–2.83)**	1.00 (0.49–2.03)	0.996
HoNOS symptoms/disorders^a^								
Agitated behaviour	1.15 (0.84–1.59)	1.12 (0.71–1.77)	0.97 (0.56–1.69)	0.918	1.09 (0.78–1.53)	0.98 (0.58–1.64)	0.95 (0.53–1.61)	0.857
Non‐accidental self‐injury	0.59 (0.29–1.19)	0.92 (0.44–1.93)	1.56 (0.56–4.31)	0.394	0.63 (0.31–1.29)	1.10 (0.48–2.51)	1.56 (0.55–4.41)	0.403
Problem‐drinking or drug taking	0.52 (0.26–1.05)	0.66 (0.28–1.57)	1.25 (0.42–3.77)	0.685	**0.43 (0.21–0.89)**	0.66 (0.26–1.72)	1.48 (0.47–4.68)	0.504
Cognitive problems	0.89 (0.69–1.16)	0.90 (0.59–1.35)	1.01 (0.62–1.63)	0.983	1.00 (0.74–1.36)	0.89 (0.52–1.54)	1.03 (0.62–1.71)	0.916
Depressed mood	0.81 (0.61–1.08)	0.89 (0.56–1.39)	1.10 (0.65–1.85)	0.733	0.81 (0.58–1.12)	0.94 (0.55–1.61)	1.12 (0.65–1.93)	0.676
Physical illness or disability	**0.68 (0.52–0.87)**	0.79 (0.51–1.21)	1.16 (0.70–1.93)	0.552	**0.69 (0.53–0.90)**	0.81 (0.50–1.31)	1.18 (0.70–1.99)	0.543
HoNOS functional problems^a^								
Activities of daily living	0.85 (0.65–1.09)	0.67 (0.43–1.05)	0.80 (0.48–1.31)	0.369	0.84 (0.63–1.10)	0.65 (0.39–1.06)	0.83 (0.50–1.40)	0.483
Living conditions	1.16 (0.80–1.68)	0.94 (0.56–1.59)	0.81 (0.43–1.54)	0.530	0.98 (0.66–1.45)	0.81 (0.46–1.44)	0.86 (0.44–1.70)	0.662
Daytime activities	1.30 (0.99–1.71)	0.75 (0.48–1.17)	**0.58 (0.34–0.96)**	**0.036**	1.25 (0.94–1.65)	0.71 (0.44–1.16)	0.60 (0.35–1.03)	0.063
Social relationships	1.31 (0.96–1.80)	1.36 (0.86–2.16)	1.04 (0.60–1.81)	0.890	1.12 (0.80–1.56)	1.19 (0.72–1.98)	1.11 (0.62–1.98)	0.734
Other contextual factors^b^								
Living alone	**2.24 (1.76–2.86)**	**1.52 (1.05–2.21)**	0.68 (0.43–1.06)	0.086	**2.47 (1.91–3.18)**	1.38 (0.93–2.07)	**0.57 (0.36–0.91)**	**0.017**
Disturbed sleep	**1.31 (1.04–1.66)**	**2.38 (1.59–3.57)**	**1.82 (1.14–2.90)**	**0.012**	1.26 (0.98–1.62)	**2.17 (1.39–3.38)**	**1.70 (1.05–2.75)**	**0.032**
Pharmacotherapy^b^								
Antipsychotic	**3.26 (2.50–4.26)**	**4.16 (2.82–6.15)**	1.28 (0.80–2.05)	0.310	**2.59 (1.19–3.49)**	**3.16 (2.06–4.83)**	1.40 (0.86–2.29)	0.177
Antidepressant	0.81 (0.64–1.03)	**0.67 (0.47–0.98)**	0.83 (0.53–1.29)	0.409	0.85 (0.65–1.10)	0.66 (0.43–1.01)	0.82 (0.52–1.30)	0.394
Mood stabiliser	**1.83 (1.10–3.04)**	**2.25 (1.19–4.26)**	1.23 (0.55–2.77)	0.617	**1.85 (1.07–3.18)**	**2.47 (1.21–5.02)**	1.29 (0.56–2.99)	0.550
Sedative medication	**1.39 (1.07–1.81)**	**2.00 (1.37–2.90)**	1.43 (0.91–2.26)	0.123	**1.44 (1.09–1.88)**	**2.09 (1.38–3.16)**	1.40 (0.87–2.25)	0.171

*Note*: Adjusted models are adjusted for age, gender, ethnicity and diagnosis; bold = *p* < 0.05.

^a^ At the time of referral.

^b^ In a 6 months' window around referral date.

## 4 DISCUSSION

In this study we investigated characteristics associated with recorded psychotic symptoms in patients referred to South London older adult mental health services during the first COVID‐19 lockdown, compared with the same period in the previous year. We found that the factors associated with any psychotic symptom in both the pre‐pandemic and first lockdown period were a diagnosis of dementia, psychotic illness, affective disorder, delirium, agitated behaviour and disturbed sleep, as well as antipsychotic and sedative use. Certain patient characteristics were more likely to be associated with psychotic symptoms during lockdown than during the pre‐COVID period. Non‐White ethnicity showed a stronger association with any psychotic symptom, hallucinations and delusions during the lockdown period. A dementia diagnosis also showed a stronger association with any psychotic symptom and hallucinations during the lockdown period, and delusions were more strongly associated with disturbed sleep during this time. On the other hand, associations of hallucinations with affective disorders and antidepressant prescribing, and of delusions with living alone, were stronger in the pre‐pandemic period.

Our finding that mental health services for older people had only 37% of the referrals during the first lockdown compared with the corresponding pre‐pandemic period in 2019 is consistent with other studies showing a decrease in secondary mental health services referrals^23^ and in mental health presentations to primary care.^24^ While the drop in referrals to MHOA services led to a reduction in caseloads, the number of discharges remained stable during the first UK lockdown. This suggests that old age psychiatry services continued to support patients already under their care, but patients with new onset mental health difficulties could have been missed.^25^ Both the reduction in presentations with milder mental health problems to primary care and barriers to accessing mental health services for older people might have resulted in proportionally more presentations with psychosis, whereby the possible reasons for this are discussed in more detail below.

The observation that a larger proportion of referrals presented with psychosis, and in particular hallucinations, could have a number of reasons. Firstly, psychosis might be more likely to emerge in older adults during a pandemic and lockdown.^14^ Various causative factors for developing psychotic symptoms during this time have been proposed, including psychosocial stress, steroid use, viral exposure, and pre‐existing vulnerabilities.^8^ Our finding of a 50% increase in hallucinations in referrals during lockdown is also consistent with reports that visual hallucinations have been one of the most common presentations to community teams for MHOA services during the COVID‐19 pandemic.^26^ These visual hallucinations might be due to more rapidly advancing dementia in the context of a lockdown, due to less access to medical care and possibly untreated infections leading to delirium, or due to a reduction in cognitive stimulating activities such as meeting friends and family.^26^ Alternatively, hallucinations could have been caused by sensory deprivation resulting from the social isolation of lockdown.^26^, ^27^ Moreover, psychotic symptoms, including hallucinations, are associated with delirium^28^ and the increase observed here could represent an increase in patients referred with delirium from all causes. It could further possibly represent withdrawal states from alcohol use, which was observed to increase early in the pandemic in the UK population, particularly in older adults.^29^

Absolute referral numbers and the proportion of patients with dementia referred to MHOA services decreased during lockdown. However, any psychotic symptoms, and hallucinations, and delusions were more likely to be associated with dementia during this period, suggesting that these symptoms played an important role in their pathway into care. Psychotic symptoms tend to increase with severity of dementia, though there are disease specific fluctuations.^30^ The association demonstrated here may reflect late presentations of dementia, caused by the reduction in access to timely dementia diagnoses and interventions, as memory services activity decreased during the pandemic.^25^ In addition, neuropsychiatric symptoms of dementia, including psychotic symptoms, are associated with carer burden and distress,^31^ and the presence of these symptoms specifically might have led to carers or patients seeking help from mental health services.

In our regression analyses, non‐White ethnicity was more frequently associated with psychotic symptoms during lockdown than during the pre‐COVID period, whereby this was more pronounced for any psychotic symptom and delusions, than for hallucinations. Ethnic minority groups have been disproportionally affected by the pandemic in terms of severe illness and mortality,^32^ which might also have an impact on their mental health. In the US, people from non‐White ethnic groups have reported experiencing significantly worse mental health outcomes,^33^ which is echoed in the UK where people from ethnic minorities have reported higher levels of mental distress during the pandemic.^34^ These higher levels of distress, in combination with an increased likelihood of a negative life event and financial concerns, are themselves risk factors for developing psychosis, and might explain the stronger associations observed in patients from non‐White groups during lockdown compared to pre‐pandemic.^9^ Additionally, evidence suggests that black older adults have fewer social relationships than white older adults,^35^ so this group may be more vulnerable to being socially isolated and lonely as result of the lockdown, both of which have been associated with psychotic illness.^36^ Furthermore, older adults from minority ethnic backgrounds experience more severe symptoms of affective illness,^37^ but are less likely to access psychological therapies or be prescribed antidepressants.^37^, ^38^, ^39^ They are also underrepresented in referrals to older people's mental health services.^39^, ^40^ This disparity seems to have been aggravated through the lockdown as in our study only 24% of referrals during lockdown were from ethnic minority groups, compared to 30% in the pre‐COIVD period. The association of non‐White ethnicity with any psychotic symptom and delusions during lockdown may represent delayed referrals to MHOA services, leading to more severe presentations of mental illness, including psychotic symptoms.

Other secondary findings from our study include the observation that patients referred during lockdown were less likely to be living alone compared with the pre‐COVID period, and that an association of living alone with delusions was less likely during lockdown compared to the pre‐COVID period. This is perhaps surprising as other studies have reported living alone as a major risk factor for developing negative mental health outcomes during social isolation.^3^, ^41^ A possible explanation for our finding is that individuals cohabiting are more likely to have family members aware of their mental health problems, will detect delusional beliefs, and encourage them to seek care. Further, lockdown was less likely to predict antidepressant use in those with any psychotic symptom or hallucinations, and less likely to predict an affective disorder in referrals with hallucinations. This might reflect a predominance of non‐affective psychosis in the referrals received during lockdown or that those with depressive psychosis might have been less easily reached/detected during the pandemic.

## 5 STRENGTHS, LIMITATIONS, AND FUTURE DIRECTIONS

To our knowledge, this is the first study analysing the impact of the first UK lockdown on factors associated with psychotic symptoms in older adults. While the main strength of our study is the use of in‐depth clinical information from a sample of older adults referred to a large mental health and dementia care provider, there a number of limitations. Firstly, the study only included referrals to specialist mental healthcare, wherein patients with mental illness of a certain severity access them and these are not necessarily comparable to patients attending primary care. Secondly, natural language processing captured the recording of psychotic symptoms in case notes, which is not the same as measuring them using a research scale. As natural language processing applications are designed to achieve a high precision (positive predictive value) at the potential expense of recall (sensitivity), there might be occasions when the psychotic symptom might not have been captured. However, as the data is from a specialist mental healthcare provider, exploration of psychotic symptoms forms is an integral part of each assessment. Relatedly, this method of capturing psychotic symptoms limits the detail available regarding the nature and degree of the symptoms, and therefore reduces the ability to associate them with specific conditions. Thirdly, the substantial decrease in the number of referrals during the lockdown period may have affected the statistical power of analyses and our ability to find potential associations in this time period group, although this does not explain the stronger associations observed for some characteristics in the lockdown period and would, if anything, obscure rather than exaggerate interaction terms. Finally, the observational nature of this study limits us from establishing causality between social distancing measures and factors associated with psychosis.

## 6 CONCLUSIONS

Our study showed that there was a higher percentage of referrals to MHOA services with any psychotic symptom and, in particular hallucinations, during lockdown. Associations of psychosis with non‐White ethnicity and a dementia diagnosis were stronger and these factors were present in a lower proportion of those referred during lockdown. This indicates that inequalities in access to care already present before the pandemic^42^ might have been exacerbated during lockdown, leading to fewer referrals and more severe presentations, especially those with disabling symptoms of psychosis. In situations when social distancing is required, older adult mental health services need to ensure access to care for people with dementia and those from ethnic minority backgrounds and should be vigilant to the potentially higher risk of presenting with psychosis in such circumstances.

## CONFLICT OF INTEREST

Robert Stewart has received research support from Janssen, GSK and Takeda. Lauren Simkin, Paul Yung, Gayan Perera, Konstantinos Tsamakis, Emmanouil Rizos, Latha Velayudhan, and Christoph Mueller declare no conflict of interest.

## Data Availability

All relevant aggregate data are found within the paper. The data used in this work have been obtained from the Clinical Record Interactive Search (CRIS), a system that has been developed for use within the NIHR Mental Health Biomedical Research Centre (BRC) at the South London and Maudsley NHS Foundation Trust (SLaM). It provides authorised researchers with regulated access to anonymised information extracted from SLaM's electronic clinical records system. Individual‐level data are restricted in accordance to the strict patient led governance established at South London and The Maudsley NHS Foundation Trust, and by NHS Digital for the case of linked data. Data are available for researchers who meet the criteria for access to this restricted data: (1) SLaM employees or (2) those having an honorary contract or letter of access from the trust. For further details, and to obtain an honorary research contract or letter of access, contact the CRIS Administrator at cris.administrator@kcl.ac.uk.

## References

[1] Banerjee D . The impact of Covid‐19 pandemic on elderly mental health. Int J Geriatr Psychiatry. 2020;35(12):1466‐1467. 10.1002/gps.5320 32364283 PMC7267435

[2] Fontes WHA , Goncalves Junior J , de Vasconcelos CAC , da Silva CGL , Gadelha MSV . Impacts of the SARS‐CoV‐2 pandemic on the mental health of the elderly. Front Psychiatry. 2020;11:841. 10.3389/fpsyt.2020.00841 32973583 PMC7461950

[3] Taylor AM , Page D , Okely JA , et al. Impact of COVID‐19 lockdown on psychosocial factors, health, and lifestyle in Scottish octogenarians: the Lothian Birth Cohort 1936 study. PLoS One. 2021;16(6):e0253153. 10.1371/journal.pone.0253153 34138930 PMC8211159

[4] Perera G , Mueller C , Broadbent M , Stewart R , Velayudhan L . Mortality among mental health services for older adults during the COVID‐19 pandemic: a retrospective analysis from South London. Int Psychogeriatr. 2021;33(5):527‐528. 10.1017/s1041610221000442 33849671

[5] Tsamakis K , Tsiptsios D , Ouranidis A , et al. COVID‐19 and its consequences on mental health (Review). Exp Ther Med. 2021;21(3):244. 10.3892/etm.2021.9675 33603852 PMC7851613

[6] Armitage R , Nellums LB . COVID‐19 and the consequences of isolating the elderly. Lancet Public Health. 2020;5(5):e256. 10.1016/s2468-2667(20)30061-x 32199471 PMC7104160

[7] Velayudhan L , Cheung WY , Perera G , Stewart R , Mueller C . The impact of the first COVID‐19 lockdown on presentations with depressive symptoms in older people ‐ an electronic health records study. Int Psychogeriatr. 2022:1‐3. 10.1017/s104161022200062x 35938206

[8] Brown E , Gray R , Lo Monaco S , et al. The potential impact of COVID‐19 on psychosis: a rapid review of contemporary epidemic and pandemic research. Schizophr Res. 2020;222:79‐87. 10.1016/j.schres.2020.05.005 32389615 PMC7200363

[9] Fusar‐Poli P , Tantardini M , De Simone S , et al. Deconstructing vulnerability for psychosis: meta‐analysis of environmental risk factors for psychosis in subjects at ultra high‐risk. Eur Psychiatry. 2017;40:65‐75. 10.1016/j.eurpsy.2016.09.003 27992836

[10] Muller F , Rohr S , Reininghaus U , Riedel‐Heller SG . Social isolation and loneliness during COVID‐19 lockdown: associations with depressive symptoms in the German old‐age population. Int J Environ Res Public Health. 2021;18(7):3615. 10.3390/ijerph18073615 33807232 PMC8036754

[11] Greig F , Perera G , Tsamakis K , Stewart R , Velayudhan L , Mueller C . Loneliness in older adult mental health services during the COVID‐19 pandemic and before: associations with disability, functioning and pharmacotherapy. Int J Geriatr Psychiatry. 2021;37(1). 10.1002/gps.5630 PMC8646648 34614534

[12] de Oliveira GC . Dementia or psychosis precipitated by social isolation? a brief case report in COVID‐19 pandemic times. Alzheimers Dement (Amst). 2021;13(1):e12166. 10.1002/dad2.12166 33855161 PMC8025932

[13] Lynch A , Bastiampillai T . COVID‐19 pandemic‐induced late‐onset psychotic depression with Catatonia. Prim Care Companion CNS Disord. 2021;23(1). 10.4088/pcc.20l02827 34000132

[14] Deshpande S , Livingstone A . First‐onset psychosis in older adults: social isolation influence during COVID pandemic – a UK case series. Prog Neurol Psychiatr. 2021;25(1):14‐18. 10.1002/pnp.692

[15] Cort E , Meehan J , Reeves S , Howard R . Very late‐onset schizophrenia‐like psychosis: a clinical update. J Psychosoc Nurs Ment Health Serv. 2018;56(1):37‐47. 10.3928/02793695-20170929-02 28990640

[16] Perera G , Broadbent M , Callard F , et al. Cohort profile of the South London and Maudsley NHS Foundation Trust Biomedical Research Centre (SLaM BRC) Case Register: current status and recent enhancement of an Electronic Mental Health Record‐derived data resource. BMJ Open. 2016;6(3):e008721. 10.1136/bmjopen-2015-008721 PMC4785292 26932138

[17] Cunningham H , Tablan V , Roberts A , Bontcheva K . Getting more out of biomedical documents with GATE's full lifecycle open source text analytics. PLoS Comput Biol. 2013;9(2):e1002854. 10.1371/journal.pcbi.1002854 23408875 PMC3567135

[18] Mueller C , John C , Perera G , Aarsland D , Ballard C , Stewart R . Antipsychotic use in dementia: the relationship between neuropsychiatric symptom profiles and adverse outcomes. Eur J Epidemiol. 2021;36(1):89‐101. 10.1007/s10654-020-00643-2 32415541 PMC7847435

[19] South London and Maudsley Biomedical Research Centre . CRIS NLP SERVICE ‐ Library of production‐ready applications Version 15; 2021. Accessed July 5, 2021. https://www.maudsleybrc.nihr.ac.uk/media/380063/applications‐library‐v15.pdf

[20] World Health Organisation . International Statistical Classifications of Diseases and Related Health Problems. 10th Revision Vol 2 Instruction Manual. World Health Organisation; 2010.

[21] Burns A , Beevor A , Lelliott P , et al. Health of the Nation Outcome Scales for elderly people (HoNOS 65+). Br J Psychiatry. 1999;174(5):424‐427. 10.1192/bjp.174.5.435 10616609

[22] Oudshoorn CGM , Buuren S , Rijckevorsel JLA . Flexible multiple imputation by chained equations of the AVO‐95 survey. TNO Prevention and Health Leiden; 1999.

[23] Chen S , Jones PB , Underwood BR , et al. The early impact of COVID‐19 on mental health and community physical health services and their patients' mortality in Cambridgeshire and Peterborough, UK. J Psychiatr Res. 2020;131:244‐254. 10.1016/j.jpsychires.2020.09.020 33035957 PMC7508053

[24] Carr MJ , Steeg S , Webb RT , et al. Effects of the COVID‐19 pandemic on primary care‐recorded mental illness and self‐harm episodes in the UK: a population‐based cohort study. Lancet Public Health. 2021;6(2):e124‐e135. 10.1016/s2468-2667(20)30288-7 33444560 PMC7843955

[25] Mueller C , Perera G , Broadbent M , Stewart R , Velayudhan L . A retrospective analysis of patient flow in mental health services for older adults in South London during the COVID‐19 pandemic. Int Psychogeriatr. 2022;34(3):1‐2. 10.1017/s1041610221002775 35001865

[26] Roufael R . Lockdown and visual hallucinations in older people: a community perspective. BJPsych Open. 2021;7(S1):S119‐S120. 10.1192/bjo.2021.348

[27] Zubek JP , Bayer L , Milstein S , Shephard JM . Behavioral and physiological changes during prolonged immobilization plus perceptual deprivation. J Abnorm Psychol. 1969;74(2):230‐236. 10.1037/h0027147 5783239

[28] Webster R , Holroyd S . Prevalence of psychotic symptoms in delirium. Psychosomatics. 2000;41(6):519‐522. 10.1176/appi.psy.41.6.519 11110116

[29] Sallie SN , Ritou V , Bowden‐Jones H , Voon V . Assessing international alcohol consumption patterns during isolation from the COVID‐19 pandemic using an online survey: highlighting negative emotionality mechanisms. BMJ Open. 2020;10(11):e044276. 10.1136/bmjopen-2020-044276 PMC7692002 33243820

[30] Aarsland D . Epidemiology and pathophysiology of dementia‐related psychosis. J Clin Psychiatry. 2020;81(5). 10.4088/jcp.ad19038br1c 32936544

[31] Baharudin AD , Din NC , Subramaniam P , Razali R . The associations between behavioral‐psychological symptoms of dementia (BPSD) and coping strategy, burden of care and personality style among low‐income caregivers of patients with dementia. BMC Publ Health. 2019;19(Suppl 4):447. 10.1186/s12889-019-6868-0 PMC6565534 31196141

[32] Kirby T . Evidence mounts on the disproportionate effect of COVID‐19 on ethnic minorities. Lancet Respir Med. 2020;8(6):547‐548. 10.1016/s2213-2600(20)30228-9 32401711 PMC7211498

[33] Czeisler ME , Wiley JF , Facer‐Childs ER , et al. Mental health, substance use, and suicidal ideation during a prolonged COVID‐19‐related lockdown in a region with low SARS‐CoV‐2 prevalence. J Psychiatr Res. 2021;140:533‐544. 10.1016/j.jpsychires.2021.05.080 34174556 PMC8177437

[34] Proto E , Quintana‐Domeque C . COVID‐19 and mental health deterioration by ethnicity and gender in the UK. PLoS One. 2021;16(1):e0244419. 10.1371/journal.pone.0244419 33406085 PMC7787387

[35] Byrne KA , Anaraky RG , Dye C , et al. Examining rural and racial disparities in the relationship between loneliness and social technology use among older adults. Front Public Health. 2021;9:723925. 10.3389/fpubh.2021.723925 34532308 PMC8438168

[36] Stilo SA , Murray RM . Non‐genetic factors in Schizophrenia. Curr Psychiatry Rep. 2019;21(10):100. 10.1007/s11920-019-1091-3 31522306 PMC6745031

[37] Vyas CM , Donneyong M , Mischoulon D , et al. Association of race and ethnicity with late‐life depression severity, symptom burden, and care. JAMA Netw Open. 2020;3(3):e201606. 10.1001/jamanetworkopen.2020.1606 32215634 PMC7325738

[38] Mansour R , Tsamakis K , Rizos E , et al. Late‐life depression in people from ethnic minority backgrounds: differences in presentation and management. J Affect Disord. 2020;264:340‐347. 10.1016/j.jad.2019.12.031 32056770

[39] Tsamakis K , Gadelrab R , Wilson M , et al. Dementia in people from ethnic minority backgrounds: disability, functioning, and pharmacotherapy at the time of diagnosis. J Am Med Dir Assoc. 2021;22(2):446‐452. 10.1016/j.jamda.2020.06.026 32758391

[40] Mukadam N , Lewis G , Mueller C , Werbeloff N , Stewart R , Livingston G . Ethnic differences in cognition and age in people diagnosed with dementia: a study of electronic health records in two large mental healthcare providers. Int J Geriatr Psychiatry. 2019;34(3):504‐510. 10.1002/gps.5046 30675737

[41] Wu B . Social isolation and loneliness among older adults in the context of COVID‐19: a global challenge. Glob Health Res Policy. 2020;5(1):27. 10.1186/s41256-020-00154-3 32514427 PMC7272234

[42] Smith S , Gilbert S , Ariyo K , et al. Multidisciplinary research priorities for the COVID‐19 pandemic. Lancet Psychiatr. 2020;7(7):e40. 10.1016/s2215-0366(20)30238-8 PMC7302780 32563315

